# Basidiospores from Wood-Decay Fungi Transform Laccase Substrates in the Absence of Glucose and Nitrogen Supplements

**DOI:** 10.3390/jof6020062

**Published:** 2020-05-14

**Authors:** Gerhard Gramss, Klaus-Dieter Voigt

**Affiliations:** 1 Institute of Geosciences, Friedrich-Schiller-University, Burgweg 11, D-07749 Jena, Germany; 2 Food GmbH Jena, Orlaweg 2, D-07743 Jena, Germany; KD.voigt@gmx.de

**Keywords:** basidiospore chemistry, biocatalysis, autonomous germination, carboxylate exudation, laccase, wood-decay fungi

## Abstract

Preparations of bacterial endospores and fungal conidia are applied in biocontrols, biocatalyses, and lignocellulose fermentations. The biocatalytic abilities of basidiospores from mushrooms of the order Agaricales are unknown. To assess their potential in colonizing recalcitrant substrates solely with their inherent resources, spores of the white-rot fungi *Stropharia rugoso-annulata* (Stru) and *Kuehneromyces mutabilis* (Kmt, Strophariaceae) were analyzed for surface-bound and internal total carbohydrates, phenols, proteins, minerals, and oxidoreductases to estimate their chemistry and the preconditions to transform the laccase substrates guaiacol and 2,2′-azinobis-(3-ethylbenzthiazoline-6-sulfonate) (ABTS) independent of external glucose and nitrogen. Surfaces of Stru/Kmt spores released (mg kg^−1^) hexoses, 7300/9700; phenols, >62/220; proteins, 21/168; and laccases, 42/0–0.15 µmol ABTS^•+^ kg^−1^ min^−1^ that mimicked oxidative activities of the resting spores. Milled-spore extracts contained pentoses, 96,600/6750; hexoses, 160,000/15,130; phenols, 452/767; protein, 12,600/924; true laccase, 688/0.30; and enzyme-protein-activating transition metals such as Cu in concentrations typical of wheat grains. Independent of external N and C supply, spores (<1‰) germinated in bideionized water, supported by their surface resources. Kmt spores germinated, too, at comparable rates in N-free solutions of glucose and the not immediately metabolizable ABTS and guaiacol. The release of proteins and oxidoreductase(s) by Kmt spores starting upon germination was higher in guaiacol-incubated idiophase- than in glucose-incubated trophophase-spores and led to the 3–4-fold formation of guaiacol polymerizates and ABTS^•+^. Constitutive aromatic ring-cleaving dioxygenases in the dormant spore that could be involved in the intrinsic metabolization of guaiacol were not detected. It is concluded that intrinsic resources enable (germinating) spores to release the highly efficient laccases of basidiomycetes and to transform aromatic compounds in the absence of sugar amendments. Spores show therefore plant seed-like autonomy in nutrient modification and acquisition during the early stages of the colonization of inert substrates.

## 1. Introduction

Commercial preparations of bacterial endospores, of conidia and spores from ascomycete fungi and yeasts, and of conidia from the basidiomycete *Peniophora gigantea* (all referred to as spores) are applied in the biocontrol of fungal and insect pests of crop plants [1,2,3] and in the control of weeds [4], the butt rot of conifers [5], and populations of (malaria) mosquitos [6,7,8]. Spore preparations of vesicular-arbuscular (VA) mycorrhizal fungi, *Rhizobium*, and *Azotobacter* spp. promote root and shoot growth in certain plant species [9,10]. Spores also serve as inocula in the large-scale fermentation of agricultural and industrial organic wastes and in the processing of lignocelluloses to value-added products [11,12,13]. As biocatalysts, spores perform redox reactions, hydrolyses, isomerizations, double-bond formations, and cleavage of C-C linkages [14]. No comparable niches have been occupied by basidiospores derived from fruitbodies of homobasidiomycetes whose basic chemistry, energy resources, and biocatalytic activities are largely unknown. Thus, their resulting degree of nutritional autonomy or dependence on external nutrients upon the colonization of recalcitrant substrates attracted no attention.

Across the fungal kingdom, spore remoistening initiates respiration and the metabolic access to internal storage compounds such as lipids [15,16], carbohydrates represented mainly by mannitol, trehalose, (cell wall) polysaccharides [17,18,19], and traces of fructose and sucrose [18]. Internal generation of, and the external supply with, D-glucose or its analogous pyranose sugars [20] provide the sources of energy [21] and the material for germination and cell wall synthesis. D-glucose is derived from D-trehalose deposits [21] and generated from acetyl-CoA, which is formed during the beta-oxidation of the fatty acids component of spore lipids. Its entry into tricarboxylic acid or the glyoxylate cycle leads to the formation of malate as another hexose precursor [16,22]. Spores also fix CO_2_ [23,24] and volatiles of short-chain alkanes [25] or microorganisms [26] to serve as sources of external carbon and of bioactive compounds. Cellulases, cutinases, esterases, and pectinases are released by the remoistened spores of plant pathogenic fungi upon leaf contacts to initiate attachment and appressorium formation [27,28,29]. Unlike the dormant and melanized basidiospore of the wood-decay fungus *Kuehneromyces mutabilis* (Schff.:Fr.) Sing. & Smith whose distinct shape is retained on outgrowth of a germ tube, basidiospores of the brown-rot fungus *Lenzites trabea* are not constitutively dormant, they germinate in the presence of a growth substrate within 4 to 5 h and turn completely into hyphae without striking changes in subcellular and cell wall organization [30,31]. The incorporation of glucose, acetate, and succinate at zero time, without any lag, suggests the presence of fully active enzyme systems in the spore. Remoistening initiates the slow formation of RNA and proteins. Growth media stimulate the synthesis of DNA and of Fe-porphyrins [31] indicative of peroxidases [32].

It is apparent that comparable metabolic activities in the pre-germination basidiospore presuppose considerable resources in storage compounds. Spores of *Agaricus bisporus* contained 17–19% lipids and 12% soluble carbohydrates in the cytosol [17]. Spores of *Ganoderma lucidum* contained 8.65% free sugars, increasing to 14.5% after hydrolysis [18]. Concentrations of protecting (phenolic) antimicrobials and reactive radical scavengers and of (enzyme) proteins and their associated minerals were not reported.

Up to 33–50% of proteins are presumably metalloproteins linked with (transition) metal cations [33,34] and comprise enzyme, transport, storage, signal transduction, and metal sensing proteins. Among intra- and extracellular enzymes from all six enzyme classes, nearly 50% of them associate with metals to function [35,36]. Upon the degradation of lignin by white-rot fungi such as *K. mutabilis*, nutrient acquisition is initiated by the consecutive one-electron oxidation (abstraction of H^+^/e^−^) from phenolic, and in concert with redox mediators, from non-phenolic lignin structures by oxidoreductase enzymes. They form the respective phenoxy and aryloxy radicals that stabilize themselves in subsequent polymerizations, disproportionations, and fragmentations of the lignin polymer. The reactions are mainly catalyzed by glycoproteins of the polyphenoloxidase (PO) complex which comprises laccases, tyrosinases, and catechol oxidases with 2–4 Cu atoms in their catalytic centers. In addition, Mn-dependent peroxidases (MnP) operate with protoporphyrin IX (Fe^3+^/Fe^4+^ redox cycle) as the prosthetic group in their catalytic center, use Mn cations as redox mediators, and are stabilized by Ca^2+^ cations (compare [32,37,38,39,40] for more details and the respective redox cycles). The expression of oxidoreductases in basidiospores of wood-decay fungi or the application of the spores as biocatalysts was apparently not investigated.

A report on extracellular laccase activities of *Trichoderma atroviride* conidia formed on a laccase-positive mycelial mat [41] prompted a test with *K. mutabilis* basidiospores, collected by placing mushroom caps in Petri dishes. Some spore prints within a dish were laccase- and MnP-negative so that spores from enzyme-positive sectors were suspect of contamination by enzymatic effluents from the cap plectenchyma but were not enzymatically active by themselves. Similar smear contact effects may add to the biocatalytic performance of many spore sources in use and support those of wood-decay fungi in their initial attack on inert natural substrates.

In this study, basidiospores of the lignocellulose degrading white-rot fungi *K. mutabilis* (6–7.5 µm long) and *Stropharia rugoso-annulata* Farlow emend Murrill (10.4–13 µm long) were collected from suspended mushroom caps to obtain free-fallen spores devoid of smear contaminations and microbial infections [42]. The in- and outside concentrations in hydrocarbons, phenols, proteins, and oxidoreductase enzymes with their associated (trace) metals were determined for the spore prints and correlated with their ability to transform the laccase substrates guaiacol and 2,2′-azinobis-(3-ethylbenzthiazoline-6-sulfonate) (ABTS) prior to germination in glucose- and nitrogen-free media and thus without the aid of external nutrients. Furthermore, their germination rates were determined in the presence of glucose, ABTS, and guaiacol as well as in their absence. The goal of this study was to assess whether the sole intrinsic storage compounds confer a plant seed-like nutritional autonomy to basidiospores in the initial establishment on recalcitrant lignocellulosics devoid of easily accessible nutrients.

## 2. Materials and Methods

### 2.1. Spore Sources

**Figure 1:**
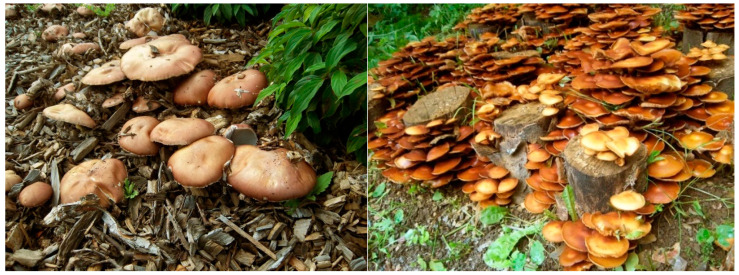
Basidiomes of *S. rugoso-annulata* (**left**) and *K. mutabilis* (Strophariaceae) (**right**).

Sporulating basidiomes of *S. rugoso-annulata* (Stru) growing on composted wood chips in the field were collected in spring and fall 2014. Basidiomes of *K. mutabilis* (Kmt) were provided by perennial field cultures established on leafwood stem segments [43] (Figure 1).

### 2.2. Spore Sampling and Washing

Mature basidiomes were fixed inside to the lids of autoclaved 0.3-L packing jars to avoid smear contaminations of the free-falling spores with plectenchyma effluents. The spores were weighed into 2-mL plastic microtubes and washed twice in 1.5-mL aliquots of bideionized water amended with the anionic detergent Fit (Fit GmbH, Hirschfelde, Germany; 5 mg per 10 mL). They were then rinsed twice in bideionized water. In a second treatment, water soluble compounds from spore surfaces were collected in 4 consecutive washings within 70 min. The spore suspensions were vigorously shaken for 10 min and centrifuged at 14,000 *g* for 5 min. Supernatants were collected for analyses.

### 2.3. Spore Incubation

Quadruplicate sets of wide-necked Erlenmeyer flasks (100 mL) were washed twice in bideionized water, filled with 6 mL of sole or combined glucose (5 g L^−1^; Merck KGaA, Darmstadt, Germany), guaiacol (133 mg L^−1^), or 2,2′-azinobis-(3-ethylbenzthiazoline-6-sulfonate) (ABTS, 224 mg L^−1^) (Fluka) solutions of highest purity (Table 1) and autoclaved at 121 °C for 30 min. To obtain surface-sterile prints of *K. mutabilis* basidiospores that were also believed to be clean of smear contaminations with enzymes from the mushroom gills, mature cap segments 2 cm in diameter were pinned inside to the cotton wool stoppers of the flasks with the gills pointing downwards [42] until rust-brown spore deposits indicated the release of 3 to 4 mg spores per flask. In a further treatment, the spore input was held below 0.3 mg. Duplicate control flasks were run without spore deposits. The samples were incubated at 15 to 19 °C in the dark until reddish-brown (guaiacol) or green (ABTS) stain reactions revealed oxidative enzyme activities. Concentrations of the respective transformation products and the current enzyme activities were determined by spectrophotometry (Helios Beta; Unicam UV-Vis; Cambridge, UK). Spore germination was followed microscopically at 2 and 7 d by retrieving 0.05-mL aliquots with aseptic pipettes.

**Figure 2:**
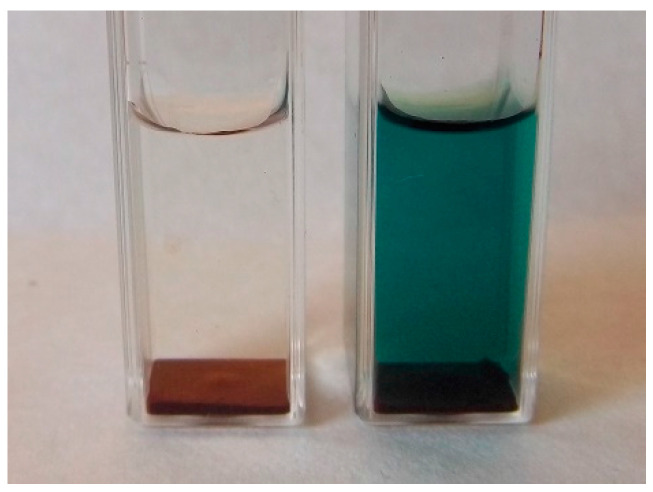
Formation of the green ABTS^•+^ by basidiospores of *K. mutabilis* in 1-cm cuvettes. Left, water control.

Using 1-cm cuvettes in preliminary assays, washed and unwashed spores were incubated in 1-mL aliquots of bideionized water, glucose, or ABTS solution under filtered-air conditions for at least 5 days. The initially hydrophobic spores sank generally to the bottom of the vessels and were not actively aerated to confine the fixation of carbon from aerial CO_2_ (Figure 2). 

### 2.4. Spore Cracking

The bullet blender PM 400 (Retsch, 42781 Haan, Germany) fitted with 20 steel balls 10 mm in diameter was used to break 250-mg samples of washed spores suspended in 1 mL of 0.1 M KH_2_PO_4_ buffer at 400 rpm over 1h. Spore fragments ≤1–10 µm were recovered with the buffer and stored at −20 °C.

### 2.5. Spectrophotometric Assays with Chromogenic Enzyme Substrates

Oxidation of ABTS by potential mixtures of laccase (EC 1.10.3.2), catechol oxidase (EC 1.10.3.1), and tyrosinase (EC 1.14.18.1) enzymes [44] in spore supernatants amended with glucose, guaiacol, or a combination of both was followed by an increase in absorbance at A_420_ (ε_420_ = 36,000 M^−1^ cm^−1^) in a reaction mixture of pH 5 containing KH_2_PO_4_ buffer (0.033 M) and ABTS (0.4 mM). Concentrations of guaiacol oligomers in spore supernatants or in separate reaction mixtures of pH 5 with 0.6 mM guaiacol were recorded at A_436_ (ε_436_ = 6400 M^−1^ cm^−1^; [45]. The oxidation of DL-DOPA (14mM) in a phosphate buffer of pH 6 was recorded as an increase in absorbance at 475 nm (ε_475_ = 3600 M^−1^ cm^−1^; [46] and confirmed by controls amended with 2 mg mL^−1^ of the tyrosinase inhibitor, gallic acid [47]. Activities of manganese-dependent peroxidase (MnP, EC 1.11.1.13) for the non-stained glucose supernatants were determined as an increase in A_265_ (ε_270_ = 11,590 M^−1^ cm^−1^) in a reaction mixture of 0.15 mM H_2_O_2_, 0.2 mM MnSO_4_ × H_2_O, and 50 mM Na-malonate at pH 4.5 [48,49]. Basidiospore homogenates were tested for activities of the aromatic-ring cleavage catalysts, catechol 2,3-dioxygenase (EC 1.13.11.2), and protocatechuate 4,5-dioxygenase (EC 1.13.11.8). More details are provided in a separate paper [50].

### 2.6. Protein and Rest Glucose

Concentrations of protein in spore supernatants were recorded at A_595_ with Bradford reagent based on Coomassie Brilliant Blue G-250 [51]. Non-consumed rest glucose was determined with Medi-Test Glucose strips (0.1 – >10 g L^−1^; Macherey-Nagel, Düren, Germany) responding to concentrations <0.01 g L^−1^ within 10 min.

### 2.7. Pentose and Hexose Sugars

Washing fluids of intact spores and supernatants of washed and cracked spores were used for the colorimetric quantitation of in- and outside pentose and hexose sugars, oligosaccharides, and their methylated derivatives [52]. A reaction mixture of 0.2 mL supernatant, 0.2 mL of a 5% (*w*/*w*) aqueous solution of phenol, and 1 mL concentrated H_2_SO_4_ was left reacting at 25 °C for 10 min. Absorbance was determined at 490 nm and compared to blank samples in which the supernatant had been replaced by water. Absorbance peaks were determined by scanning spectrophotometry to discriminate between peaks at 480 nm (for pentoses, methylpentoses, uronic acids), and 485 to 490 nm (for hexoses and their methylated derivatives). Calibration was performed with 10, 50, and 150 mg L^−1^ aqueous xylose and sucrose solutions, respectively.

### 2.8. Total Phenols

Fast Blue B salt (tetrazotized *o*-dianisidine, Acros, Geel, Belgium) reacted with 1-naphthol and other hydroxylated aromatic compounds to form coloured products whose maximum absorbance was in the range 530 to 618 nm [53]. In adaptation, spore supernatants of 0.3–1 mL were filled up to 1.9 mL with 0.2 M KH_2_PO_4_ buffer pH 4.5 in 1-cm glass cuvettes by determining their absorbance correction values at A_530_. Then 0.1 mL of a KH_2_PO_4_-buffered and freshly prepared Fast Blue B solution (10 mg mL^−1^) was added and shaken to record the rapidly increasing absorbance value at 10 s. Results were expressed in pyrogallol equivalents.

### 2.9. HPLC-MS Analysis of Guaiacol Transformation Products

Oligomers in guaiacol-amended spore supernatants were analyzed with an Agilent 1100 HPLC (Agilent Technologies) equipped with a Bruker Esquire 6000 Ion Trap Mass Spectrometer detector (Bruker Daltonics, Bremen, Germany) and a Nucleodur Sphinx RP 5 μm column (250 × 4.6 mm, 5 μm, Macherey-Nagel, Düren, Germany). The solvent systems comprised 0.2% formic acid in water (A) and acetonitrile (B) used in gradient mode at a flow rate of 1 mL min^−1^ at 25 °C. The gradient was as follows: 100% A (5 min), 0–75% B (25 min), 75–100% B (0.1 min), 100% B (4.9 min), and 100% A (4.9 min). The mass spectrometer was operated in an alternating ionization mode in the range m/z 60–1400. Skimmer voltage, ±35 eV; capillary exit voltage, ±102 eV; capillary voltage, ±4000 V; nebulizer pressure, 35 psi; drying gas, 11 L min^−1^; gas temperature, 330 °C.

### 2.10. HPLC-MS/MS Analysis of Carboxylic Acids

Carboxylic-acid exudates of submerged and non-germinated spores were analyzed with a Shimadzu LC-20 AD HPLC with API 4000 mass spectrometer (Applied Biosystems) and Phenomenex Luna 3µ PFP (2) 100A column 150 × 3.00 mm. The mobile phases used in gradient mode comprised the solvents A (water: methanol 99:1 with 5 mM ammonium acetate) and B (methanol: acetic acid 99:1 with 5 mM ammonium acetate). Aliquots of 5 µL were analyzed within 15 min at 30 °C of column temperature.

### 2.11. Spore Mineral Content

Duplicate 300-mg samples of unwashed basidiospores were microwave digested in HNO_3_ (4 mL) and H_2_O_2_ (1 mL) and diluted to 150 mL with bi-deionized water. The solutions were analyzed by Inductively Coupled Plasma Mass Spectrometry (ICP-MS; Thermo, X series, Thermo Fisher Scientific, Dreieich, Germany). Argon was used as the carrier gas. One-point calibration was done with the metal standard solutions, Merck VI and Merck XXI (Merck, Darmstadt, Germany), diluted to 5%. Resulting detection limits in mg kg^−1^ dry weight (DW) were as follows: 0.0005, Th; 0.002, CdCoCsU; 0.005, Cr; 0.01, AsMnPb; 0.02, CuSr; 0.03, BaNiZn; 0.04, Fe; 0.1, AlMg; 0.4, KNa; 1, P; and 3, Ca.

### 2.12. Statistical Treatments

SPSS 8.0 software (SPSS Inc., Chicago, IL, USA) was used to calculate standard deviations and confidence levels of duplicate to quadruplicate results from two to four replicates of the individual experiments.

## 3. Results

### 3.1. In- and Outside Concentrations of Major Organic Spore Constituents

The large resting spores of Stru released surprising activities of laccase, high concentrations of carbohydrates, little phenolics, and non-significant traces of protein into the first washing fluid (Table 2). Apart from surface phenolics, most constituents declined to marginality in the fourth wash. Internal concentrations in carbohydrates >> protein >> phenolics and laccase surpassed the outside resources by more than an order of magnitude. Reaction with the p-diphenol and syringaldazine identified oxidoreductase as the true functional endolaccase [54]. Chromogenic tests for internal tyrosinases and Mn peroxidases were negative. The small melanized spores of Kmt released some carbohydrates, protein, and almost non-removable stocks of phenolics into the yellowing first wash, completed by negligible-to-lacking traces in surface laccase (Table 2). Similarly, most of these compounds from inside the spores lagged far behind those found in Stru which applied in particular to the negligible endolaccase activities.

**Figure 3:**
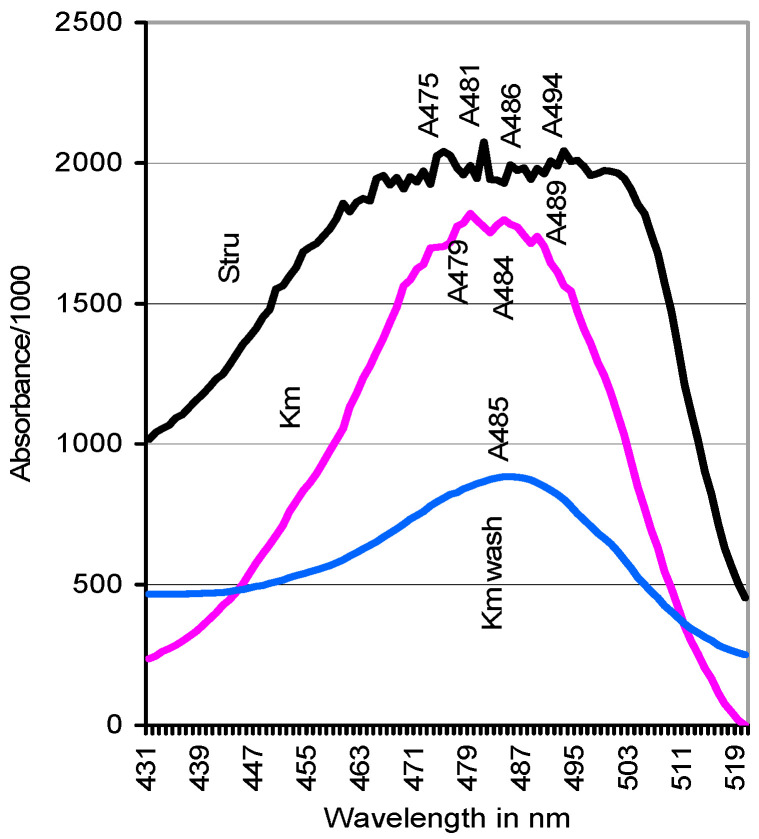
Absorbance spectra indicative of the expression of pentoses and hexoses in the internal carbohydrate pool of washed *S. rugoso-annulata* and *K. mutabilis* spores and in the first wash of Kmt (A_485_).

Absorbance spectra of carbohydrates displayed a single peak for hexose sugars at A_485_ for the first wash of both spore sources (Figure 3; compare Section 2.2). Internal carbohydrates of washed Stru spores showed uniformly high absorbance values from A_475_ to A_480_ for pentoses which passed over to A_485_ to A_494_ values for hexoses. Those of Kmt spores formed single peaks at A_479_ and A_484_.

### 3.2. Concentrations of Enzyme-Associated, Essential Minerals in Spores

Table 3 presents the outregulated metal load of wheat grains as the only available landmark for tolerable concentration spans which ensures, e.g., the adequate supply of the germinating seedling by avoiding metal stress [55,56]. Unwashed spores of Kmt as organisms adapted to the same geochemical environment were moderately higher in Co, Fe, K, Mn, Na and Ni but not in the other minerals.

Of the metals present in higher numbers of enzymes, Fe and Mn as the co-factors of fungal class II peroxidases but not Mg and Zn clearly surpassed the concentration limits known from grain crops (Table 3). Contemporarily, the unwashed Kmt spores released 55%/13%/2.8%/3.4% of their total Ca, Cu, Fe and Mn resources into bideionized water (Table 1). Unwashed spores of Stru were considerably higher in the enzyme-associated Fe and Na and moderately elevated in Ca and Co but did not leave the concentration range of grains in regard to the other essential elements (Table 3).

### 3.3. Basidiospore Germination under Nutrient-Limited Conditions in 1-cm Cuvettes

The 4-mg samples of washed and unwashed basidiospores deposited at the bottom of cuvettes (Figure 2) started germinating late at the second day in a 5‰ glucose solution (1 mL) of high purity (Table 1) but not yet in the bideionized water. At 5 d, both the spores of Stru and Kmt had formed vertical hyphae on the bottom deposits as well as hyaline floating clusters of spores connected by clamped hyphae. Further spores formed germ tubes of 2.5 to 25 µm in length. Whereas the germination rates were estimated to amount to 35%, the use of unwashed spores led to the formation of the 4- to 5-fold quantity of visible hyphae (Table 4). Of the spores of Stru suspended in bideionized water, <1‰ formed stagnating hyphal tips of 2.5 µm at the germ porus, with a significantly higher rate in the unwashed treatment. Similarly, unwashed Kmt spores developed the hyphal tips at an extremely lower percentage while washed spores failed to germinate (Table 4).

### 3.4. Release of Carboxylic Acids by Pre-Germination Spores in Cuvettes

**Figure 4:**
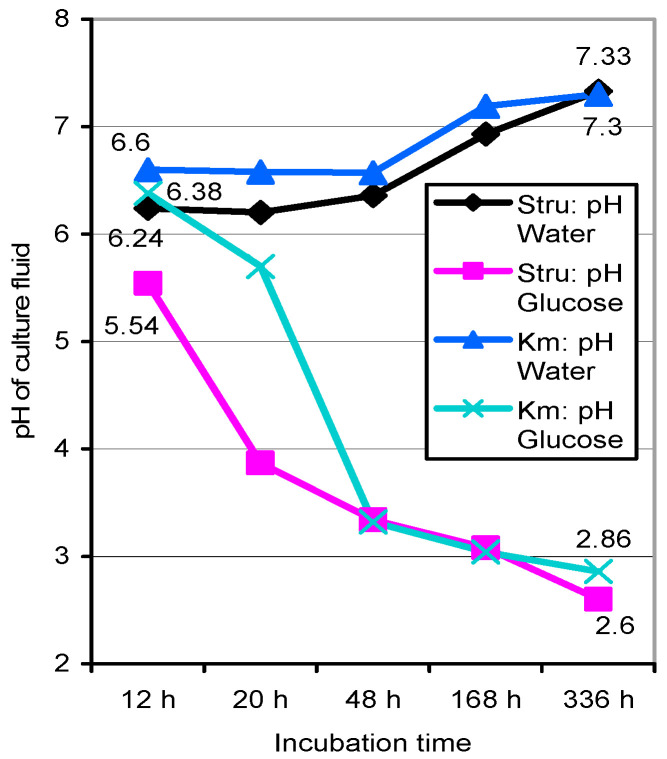
pH shifts in aqueous and glucose-amended 1-mL suspensions of unwashed *S. rugoso-annulata* and *K. mutabilis* basidiospores (4 g L^−1^) incubated in cuvettes over 336 h. Values deviate <0.1 to 0.2 pH units from those of washed basidiospores.

Incubating 4-mg samples of unwashed spores in 1-mL aliquots of bideionized water or a 5‰ glucose solution of high purity as above led to the acidification of the latter by non-germinated Stru > Kmt spores even within 12–20 h (Figure 4). Observation of the cultures over 14 d found the spores of Stru/Kmt united at pH 2.6/2.86 in glucose solution and alkalinized at pH 7.3 in water.

Incubation of spores with the pure guaiacol solution in 1-cm cuvettes did not result in significant pH changes. Washed Stru spores acidified glucose medium with the aliphatic carboxylates citrate, fumarate, malate, and succinate (Table 5) which are typical of tricarboxylic acid and the glyoxylate cycle [22]. The early presence of glycolate, malonate, and tartrate results from the respective enzymatic derivations. Traces of the aromatics, benzoic, and gallic acid were also found. They appeared at lower concentrations in the first wash of Stru spores, too, combined with succinate (Table 5).

### 3.5. Oxidation of Guaiacol by Basidiospore Suspensions of K. mutabilis in Erlenmeyer Flasks

Activities of the aseptic, unwashed, and exolaccase-deficient Kmt spores (Table 2) discharged immediately into the N-free and high-purity glucose and guaiacol media (Table 1 and Table 6) were followed by analyzing 0.3-mL aliquots. No signs of germination and significant exoenzymatic activities were recorded at 2 d. Germ tubes 15 to >35 µm long in <1‰ of the spores had developed at 7 d in the glucose, guaiacol-only, and glucose/guaiacol media but dropped to a visually estimated 10% in the presence of ABTS. The contemporary production of the reddish-brown guaiacol transformation products peaking in absorbance at 470 nm seemed to slow down to a temporary plateau concentration from 7 to 11 d. Their concentration in the guaiacol-only treatment surpassed that in glucose/guaiacol medium by a factor of four (Table 6). Accordingly, activities of polyphenoloxidases expressed in the ABTS assay and the respective release of (enzyme) proteins were greatly stimulated by guaiacol and apparently repressed by the poorly consumed glucose. The solubilized protein reached the 5-fold of the 1.1 g kg^−1^ found inside and outside the resting spore itself (compare Table 2).

Comparable quantities of stunting mycelia represented by clusters of germinated spores were formed in the presence of glucose as well as of guaiacol-only (Table 6). The acidification of the incubation media to pH values of 2.6 to 2.8 that was started by non-germinated spores within 0–12 h (Figure 4) proceeded both in the presence of external glucose as well as pure guaiacol. The apparent spore-internal perception and metabolization of guaiacol should commence with the cleavage of its aromatic ring. Activities of the respective dioxygenases in milled homogenates of both spore sources, however, could not be shown.

### 3.6. Oxidation of ABTS by Basidiospore Suspensions of K. mutabilis in Erlenmeyer Flasks

In reference to the control solution of pure ABTS, the small spore lots of <0.3 mg (0.5 g L^−1^) suspended in ABTS-only and glucose/ABTS solutions did not raise the absorbance at A_420_ over those in the controls by 6 d and displayed no PO activities from the spore surface. At 9 d, one sample of the quadruplicate glucose/ABTS treatment had oxidized 66.7 of the 408 µM ABTS provided (Table 6) and harboured sparse clusters of germinated spores. At 12 d, spore germination in the presence of ABTS-only and glucose/ABTS was common, with the production of ABTS^•+^ stagnating at 1.9–67 µM to the advantage of glucose-containing samples (Table 6). This early plateau formation in the oxidation of ABTS to ABTS^•+^ was not due to its further conversion to the red-coloured ABTS dication (ABTS^2+^) [58] or to the final degradation of the ABTS molecule itself [59].

In two samples of a quadruplicate control treatment, the sparsely germinated spores had formed mats of aerial mycelium up to 10 × 14 mm on pure glucose medium at 38 d. The samples displayed the outstanding enzymatic activities of 2.3 and 6.1 µM min^−1^ in the ABTS assay (Table 6).

### 3.7. HPLC-MS Analysis of Guaiacol Oligomers in Cuvette Cultures of K. mutabilis Spores

In re-examining laccase-catalyzed guaiacol (M 124.14) transformation products, 12 mg aseptically collected spores were incubated in 2 mL of guaiacol-amended 0.05 M KH_2_PO_4_ buffer pH 5 (6 g L^−1^ of spores) under filtered air conditions. A_470_ absorbance values reached 0.157 in 8 d of incubation. Mass spectral analyses indicated the presence of a spore-catalyzed guaiacol dimer (M 246) and a trimer (368) of two tetramers (490) distinguished by retention times of 27.8 and 28.6 min, and of a dimeric orthoquinone (260) (compare [60,61,62,63] for structural analogues). Unique compounds of MW 206 and 298 also appeared in notable concentrations. Tetraguaiacol (488) consisting of two C-O-O-C coupled dimers and immediately metabolizable guaiacol moieties <M 124.14 were not detected.

## 4. Discussion

### 4.1. Organic Resources and Protective Substances of Basidiospores

Apart from the ubiquitous lipids [16,17], intrinsic carbohydrates were the major energy resources of spores determined to act in solutions of poorly metabolizable chemicals such as guaiacol and ABTS. They reached 160/15 g kg^−1^ sucrose and 97/6.8 g kg^−1^ xylose equivalents in spores of Stru and Kmt, respectively (Table 2). Surprising amounts of 7.3/9.7 g kg^−1^ hexose sugars (Figure 3) corresponding with 4/39% of the total content were washed from outside the free-fallen spores and could possibly not be rated as accidental smear contaminants. The concentrations ranged in the order of those given for basidiospores of *Agaricus bisporus* [17] and *Ganoderma lucidum* [18]. Intrinsic soluble Bradford proteins [51] amounted to 12.6/0.9 g kg^−1^ of dry Stru/Kmt spores (Table 2) and differed apparently from the cell-wall bound compounds that respond in spores of *Pisolithus microcarpus* to staining [19]. In herb tissues, Bradford-protein values as reported in this study reached hardly 25% of those determined by the Kjeldahl method (unpublished). Spore external proteins were negligible.

Few reference values have been reported for spore-internal soluble phenols (Table 2) which may help in preserving the spore integrity in the field and on exposure to chemicals in biocatalytic applications. In spores of *P. microcarpus*, the presence of phenols could not be ascertained by staining [19]. From spores of *A. bisporus*, *p*-coumaric and the non-phenolic cinnamic acid were extracted [18]. In the spores of Stru and Kmt, four-step washings could not overcome the persistence of external phenolics (Table 2) as they may be part of the polyphenolic spore coat melanins which protect against oxidants, free radicals, and UV and gamma irradiation [64,65]. Phenolics are common to plants. Mycelia and sporocarps of most wild mushrooms are sources of hydroxylated benzoic and cinnamic acid derivatives, flavonoids, and tannins e.g., [66,67,68] with variable antibacterial properties [66]. They are constitutive antimicrobials and (oxygen) radical scavengers or are formed in response to wounding and pathogen ingress [69]. Cultivated mushrooms contained 31–63/0.05–1.57 mg kg^−1^ DW in the gallic/benzoic acids [70] found in spores of *S. rugoso-annulata* (Table 5). Total phenols in sporocarps ranged 2.8–6.3 g kg^−1^ DW [71] and surpassed the spore-typical concentrations.

### 4.2. Polyphenoloxidases and Their Metal Co-Factors in Resting Spores

True functional endolaccase was the sole polyphenoloxidase from inside the milled spores and was surprisingly high in those of Stru and marginal in those of Kmt (Table 2). Intrinsic tyrosinases and peroxidases for potential biocatalytic reactions were not detected. Exolaccases at 6% of the total activity could even be washed from free-fallen spores of Stru but virtually not from those of Kmt. Laccase may contribute to melanin synthesis [72,73] and commonly to the pigmentation of ascomycete conidia [74,75]. Laccase glycoproteins are also structural components of endospore coats in *Bacillus subtilis* [73,76]. This does not seem to apply to spores of Stru. The rapid downwash of the proteins (Table 2) points to constitutive or accidental surface coatings which mimic laccase activity of the resting spore.

The metallome of Kmt basidiospores was actively adapted [43] to the concentration spans which ensure the vigorous and metal stress-free development of seedlings in seed crops [55,56] with which fungi share the common geochemical environment. Up to 55/13% of the total Ca/Cu stock was located on the spore surface (Table 1 and Table 3). The spores’ mineral stock could thus be high enough to equip their enzyme proteins with metal cofactors and to ensure their independence of external minerals during the initial metabolic steps in high-purity solutions (Table 1). Unwashed spores of Stru combined 20 mg kg^−1^ Cu (0.32 mmol) (Table 3) with 12.6 g kg^−1^ of intrinsic soluble protein (Table 2). An equimolar Cu concentration would come to 0.1575 mmol if the entire protein would be made up of sugar-free laccase M 80,000. To equip the protein with the fourfold molar concentration of its metal cofactor, 0.63 mmol Cu cations had to be provided. This means in turn that the small (glyco)protein fractions of endolaccases in both spore sources should be saturated by the internally available catalytic Cu cations compare [32,37,38,39,40] to ensure their functionality.

### 4.3. Early Metabolic Activities of Basidiospores and Their Partial Germination

Exuded aliphatic and aromatic carboxylates determined for the unwashed and non-germinated spores of Stru in 1-cm cuvettes (Table 5) acidified a glucose medium by 0.7 pH units even within 12 h of imbibition and reached values of pH 2.6 (Figure 4). Spore incubations in water or the guaiacol solution did not result in pH drops. In contrast, unwashed Kmt spores acidified their media in the presence of glucose, glucose/guaiacol, or the not immediately metabolizable guaiacol itself (Table 6, Figure 4). The release of carboxylates apparently by both non-germinated spore sp. at zero time thus seemed to be linked with perception or uptake of (potential) external nutrients at least in regard to glucose. This indicated the early functionality of the enzyme systems involved in tricarboxylic acid and the glyoxylate cycle and in the subsequent enzymatic derivation of their primary carboxylates to glycolate, malonate, and tartrate in the formation of further carbon skeletons for amino acids [22].

The slight pH increases in water as incubation medium (Figure 4) are reminiscent of alkalinations caused by H^+^ consuming decarboxylations of (spore external) carboxylic acids (Table 5) [77] and the generation of OH^−^ during their oxidative degradation [78,79]. The release of internal and external aromatic carboxylates from the stock of total phenols (Table 2 and Table 5) may be accidental. Gallic acid with its moderate antimicrobial activity is more notorious for its inhibitory effects on laccases > tyrosinases [80]. It is transformed to pyrogallol by gallate decarboxylase [81] or oxidatively degraded to aliphatic carboxylates by aromatic-ring cleavage [82] to enter the tricarboxylic acid cycle. The early release of carboxylate ligands active in the mobilization of essential trace minerals [83] and active in the fragmentation of soil humic colloids into metabolizable units [84] confers considerable ecological advantages to the pre-germination spore.

In the glucose solution, the production of germ tubes and floating mycelia represented by germinated-spore clusters from days 3 to 5 was 4–5 times as high in unwashed than in washed spores (Table 4), although their germination rates were congruent with the estimated 35%. In water, spore external compounds such as the 7.3–9.7 g kg^−1^ hexose sugars corresponded with a concentration of 0.04 g L^−1^ in the incubation medium (Table 2). This improved the minor germination rate of <<1‰ in both spore sp. visibly (Table 4). Washed spores at least of Stru germinated principally if set back solely to their internal resources. Some nutritional support by aerial CO_2_-carbon [24,31] should not be excluded.

### 4.4. Expression of Exo-Oxidoreductases Was Confined to the Germinating K. mutabilis Basidiospore

The exolaccase deficient Kmt spores in Erlenmeyer flasks at 0.5–0.67 g L^−1^ formed comparable quantities of germinated-spore clusters on N-free glucose, pure guaiacol, and glucose/guaiacol media between 2 to 11 d and acidified their incubation media to the same extent by the apparent release of carboxylates starting almost at zero time (Table 6, see above). Contemporarily, the oxidation of guaiacol commencing with spore germination, the appearance of exo-polyphenoloxidase activities, and the exudation of (enzyme-glyco-) proteins was higher in the pure guaiacol treatment and apparently inhibited by glucose (Table 6). Exuded proteins up to 4.4–5.3 g kg^−1^ surpassed the load of 0.168 g kg^−1^ retrievable from outside the spores themselves considerably (Table 2). The presence of non-consumed glucose at 2.5–3.8 g L^−1^ in the incubation media makes spores persist in the trophophase state which is denoted by a significantly repressed laccase activity. The transition from fungal tropho- to idiophase with the depletion of internal and the absence of external carbon leads to the enhanced formation of laccase [85,86] due to the outset of fungal proteolysis [86] and the production of secondary metabolites [87].

Spore stocks < 0.05 g L^−1^ germinated, too, on a pure solution of the heterocyclic ABTS whose oxidative transformation ended in the state of ABTS^•+^ to indicate that its C, N, and S constituents did not immediately contribute to spore nutrition (Table 6). Nevertheless, germination of the submerged spores and the enzymatic transformation of guaiacol and ABTS came finally to a standstill in all treatments within 12–25 d and may indicate the depletion of the spores’ non-structural N stock. Furthermore, pH conditions between 2.6–2.8 may not only be detrimental to spore germination. It was shown that pH values between 2.1–2.2 deactivated Kmt laccase completely within 4–5 d [88]. In the case of Kmt spores that formed aerial mycelia on the surface of glucose solution, access to air resulted in higher pH conditions and an enhanced production of oxidoreductase enzymes (Table 6) that seemed to comprise tyrosinase and MnP activities typical of Kmt mycelia [89].

Kmt spores incubated submerged in non-glucose media of Erlenmeyer flasks did not display energy deficits in spite of their apparent idiophase state. Guaiacol molecules (M 124.14) smaller than those of glucose (M 180.16) should readily be taken up but should not enter the energy generating pathways prior to aromatic-ring cleavings. The respective spore internal enzyme systems of the *meta*-cleavage pathway, catechol 2,3- and protocatechuate 4,5-dioxygenase [80], were not constitutively expressed in the spore homogenates. In reactions catalyzed by lignin peroxidase, incorporation of O_2_ from H_2_O by radical reactions could also result in cleavages of aromatic rings [90]. Spore-external activities represented mainly by laccase-like enzymes caused guaiacol molecules exclusively to polymerize (Section 3.7) after the typical one-electron oxidations [32,37,38,39,40] to block their immediate metabolization. Dioxygenases of the *meta*-cleavage pathway were generally wide-spread in outdoor fungal mats [50] and their formation by the germinating spore should not be excluded. Consumption of the aromatic compound could thus contribute to the higher activities of guaiacol versus guaiacol/glucose incubated spores (Table 6).

Beneficial effects of ABTS on spore metabolic activities remain obscure. The compound of M 548.68 is oxidized by laccases (and peroxidases) such as above to the stable ABTS^•+^ by one-electron abstraction. Its subsequent oxidation to ABTS^2+^ by the enzymes alone is controversial [91,92]. It was, however, shown that enzyme-free ABTS^•+^ preparations are reduced to ABTS during their abiotic oxidation of low-MW extractives from lignocelluloses (and of other organic traces) to redox mediating radicals. The radicals, in turn, transform the remaining ABTS^•+^ to the red ABTS^2+^ compound, to red protein/ABTS adducts, and to further ABTS moieties [93]. The dication is accessible to slow decay by inorganic peroxides [64] but may end in red azo dye fragments [93].

## 5. Conclusions

Washings revealed the non-existence of the expected “externally clean” free-fallen spores. Beside the antimicrobial and structural phenolics, several surface carbohydrates, proteins, minerals, and aliphatic and aromatic carboxylates were washed off. Surface exolaccase mimicked oxidative activities of the resting spores. Intrinsic constituents enabled washed basidiospores of *S. rugoso-annulata* to form stagnating germ tubes at a rate of <1‰ in bideionized water. Germination was promoted by their surface constituents. Spores of *K. mutabilis* showed comparable rates of germination (<1‰) on N-free incubation media with glucose or the not immediately utilizable aromatic/heterocyclic substances, guaiacol and ABTS in standing liquid culture and acidified the media to pH 2.6–2.8 apparently with carboxylic acids that are known to mobilize minerals and organic nutrients from soil humic substances. With the formation of extensive germ tubes, proteins and functional polyphenoloxidase(s) derived from intrinsic deposits of nitrogen and reactive Cu cations were released. The dominating true laccase then oxidized ABTS to the temporary dead-end product ABTS^•+^ and formed polymers, but no low-MW fragments from guaiacol. It is postulated that the formation of first mycelia and the resulting release of oxidoreductases proceeded with the spores’ internal and surface-bound resources. This confers some plant seed-like autonomy to basidiospores of wood-decay fungi in the initial attack on lignocelluloses devoid of easily accessible N and C sources. This can be seen as an ecological and competitive advantage in the colonization of poor substrates in the field. The results open chances especially for idiophase spores to transform or (co-)polymerize those aromatic chemicals in liquid state fermentations (and in the absence of sugars) which are substrates of the highly efficient oxidoreductase enzymes of basidiomycetes. Therefore, commercially available spores of the indoors cultivated mushrooms *Agaricus bisporus* and *Pleurotus ostreatus* will be subjects of further studies into potential biocatalytic applications due to their year-round availability.

## Figures and Tables

**Table 1:** Mean concentrations (µg L^−1^ ± SD; *n* = 2) in the high-purity incubation media of metal cations which are commonly associated with laccase and MnP metalloenzymes (ICP-MS data).

Medium	Ca	Cu	Fe	Mn
Bideionized water	<10	0.39 ± 0.12	0.12 ± 0.04	0.01 ± 0.01
Glucose 5 g L^−1^	34 ± 7	1.3 ± 0.32	0.27 ± 0.08	0.13 ± 0.04
Guaiacol 133 mg L^−1^	34 ± 4	1.4 ± 0.12	0.45 ± 014	0.08 ± 0
ABTS 224 mg L^−1^	64 ± 1	1.8 ± 0	0.61 ± 0.02	0.34 ± 0.06
Spore suspension ^a^	111 ± 16	2.4 ± 0.92	3.8 ± 1.2	1.66 ± 0.12

^a^ Unwashed and free-fallen Kmt basidiospores (0.67 g L^−1^) infused in bideionized water for 2 d released thus (mg kg^−1^ spores): Ca, 152 ± 28; Cu, 3 ± 0.9; Fe, 5.5 ± 1.3; Mn, 2.5 ± 0.13.

**Table 2:** Laccase activity (µmol ABTS^•+^ kg^−1^ min^−1^ ± SD), and the concentrations of protein, carbohydrate, and total phenol (mg kg^−1^) in the four consecutive washing fluids and inside the washed and milled basidiospores of *S. rugoso-annulata* and *K. mutabilis* (*n* = 2–4).

Species	Extract	Laccase	Protein	Carbohydrate	Total Phenol ^c^
*Stropharia*	1. Wash	32.8 ± 3.90	21 ± 99	7270 ± 33 ^a^	18.5 ± 2.1
	2. Wash	7.38 ± 2.56	ND	ND	18.8 ± 3.6
	3. Wash	1.34 ± 1.34	ND	ND	13.3 ± 1.8
	4. Wash	0.28 ± 0.20	ND	ND	10.9 ± 0.1
	Total 1–4	41.7 ± 4.9	21 ± 99	7270 ± 33 ^a^	61.5 ± 4.6
	Inside concentr.	688 ± 84	12,600 ± 405	160,000 ± 2670 ^a^ 96,600 ± 1200 ^b^	452 ± 53
*Kuehneromyces*	1. Wash	0.15 ± 0.15	168 ± 121	9730 ^a^	68.3 ± 6.3
	2. Wash	ND	ND	ND	73.6 ± 6.9
	3. Wash	ND	ND	ND	36.9 ± 2.5
	4. Wash	ND	ND	ND	40.2 ± 0.5
	Total 1–4	0.15 ± 0.15	168 ± 121	9730 ^a^	219 ± 9.7
	Inside concentr.	0.30 ± 0.03	924 ± 49	15,130 ± 490 ^a^ 6750 ± 56 ^b^	767 ± 27

^a^ Expressed in sucrose equivalents; ^b^ in xylose equivalents; ^c^ in pyrogallol equivalents. ND, not detected.

**Table 3:** Out-regulated concentrations (mg kg^−1^ ± SD by dry weight (DW)) of essential, enzyme-asssociated minerals in unwashed basidiospores of *S. rugoso-annulata* and *K. mutabilis* in comparison to those in wheat grains (*n* = 2).

Element	*Stropharia*	*Kuehneromyces*	Presence in Enzymes ^a^	Concentration Ranges in Whole Wheat Grains ^b^
Ca	755 ± 44	277 ± 19	2	280–540
Cd	0.860 ± 0.012	0.850 ± 0.003	(1 enzyme)	0.02–3 (0.05–0.4)
Co	0.094 ± 0.002	0.705 ± 0.006	1	<0.03
Cu	20.3 ± 0.3	24 ± 0.6	1	11–14 (2–20)
Fe	385 ± 5	199 ± 4	8	40–64
K	6200 ± 42	8160 ± 32	0.5	4100–6500
Mg	640 ± 4	1140 ± 3	16	1160–1700
Mn	28 ± 0.2	73.5 ± 1.2	6	18–34 (14–30)
Na	304 ± 4	92 ± 6	0.5	30–50
Ni	3.43 ± 0.32	13 ± 0.07	0.5	0.18–1.4 (0.1–3)
P	5470 ± 64	5150 ± 43		4000–5300
Zn	79 ± 2	75 ± 0.8	9	35–190 (10–100)

^a^ Percentage of enzymes from all six enzyme classes with which the metal cation associates [36]. ^b^ Adapted from Gramss and Voigt [43,56]. In parentheses, normal spans of mineral concentrations in dry food crops [57].

**Table 4:** Comparative germination of unwashed and washed basidiospores (4 g L^−1^) in 1-mL aliquots of glucose solution (5 g L^−1^) and bideionized water in 1-cm cuvettes at 23 °C for 5 d (*n* = 3–5). Mrel, visually estimated relative mycelial quantity.

Medium	Treatment	*Stropharia*	*Kuehneromyces*
Glucose solution	Unwashed	On spore deposits, vertical hyphae 1.5–3 mm, and floating mycelia up to 3 mm; Mrel = 1	On spore deposits, vertical hyphae 1–1.5 mm, and floating mycelia up to 1.5 mm; Mrel = 1
	Washed	Vertical hyphae 0.7 mm, no floating mycelia; Mrel = 0.2. Both germination rates around 35%; rest glucose, 4.5 g L^−1^	Vertical hyphae 0.5–1 mm and traces of floating mycelia; Mrel = 0.25. Both germination rates around 35%; rest glucose, 4.5 g L^−1^
Water	Unwashed	<1‰ of spores with germ tubes 2.5 (to 25) µm; Mrel = 1	<<1‰ of spores with germ tubes 2.5 µm
	Washed	<1‰ of spores with germ tubes 2.5 (to 25) µm; Mrel = 0.6	No germinating spores

**Table 5:** Carboxylic acids (mg kg^−1^ spores, *n* = 1) released by unwashed and non-germinated spores of *S. rugoso-annulata* (6.7 g L^−1^) in glucose solution pH 2.71 within 60 h (rest glucose, 4.7 g L^−1^) and into a pool of five first washing fluids.

Carboxylic Acids	In Glucose Solution	In the First Wash
Aliphatic acids		
Citric	766	ND
Fumaric	1340	ND
Glycolic	150	ND
Malic	1800	ND
Malonic	1340	ND
Succinic	8415	230
Tartaric	105	ND
Aromatic acids		
Benzoic	<15	4.4
Gallic	<15	<5.6

ND, not determined.

**Table 6:** Activity ranges of unwashed and aseptically germinating *K. mutabilis* basidiospores in 6 mL of high-purity solutions of glucose, guaiacol, and ABTS determined at 11 to 12 d (*n* = 3–4).

Spore Incubation Medium Amended with (L^−1^)	Relative Mycelial Quantity	Plateau Concentr. of Product µM	Enzymatic ABTS Oxidation in µM Per53 h ^b^ Per Min ^a^		Protein Released g kg^−1^ Spores	Rest Glucose g L^−1^	Mean Final pH of Spore Medium
Spore load 3–4 mg per flask (0.5–0.67 g L^−1^)							
Glucose 5 g	1		3.4–35		0.6–1.7 ^2^	2.5–3 ^2^	2.6
Guaiacol 133 mg	1.3	64–133 ^1^		0.7–1.3 ^1^	4.4–5.3 ^1^	0 ^1^	2.8
Glucose/guaiacol	0.8–1.7	16–25 ^2^	6.6	0.14 ^2^	1.8–3.0 ^2^	3.5–3.8 ^2^	2.7
Spore load < 0.3 mg per flask (<0.05 g L^−1^)							
ABTS 224 mg	<0.1	1.9–47	ND	ND	ND	0	
Glucose/ABTS	<0.1	2.3–67	ND	ND	ND	4.5–5	
Glucose 5 g	2 ^c^			2.3–6.1 ^c^			3.8 ^c^

^a^ Enzyme activities (µM min^−1^) are re-calculated for 100% of spore incubation medium in the reaction mixture. ^b^ Values obtained with 0.33 mL of spore incubation medium in 1 mL of reaction mixture. ^c^ Mycelium with aerial hyphae on the incubation medium. Data ranges with different superscript numbers in the guaiacol treatments differ significantly at *p* ≤ 0.05. Detection limits, 0.3–0.5 µM for guaicol and 56–83 nM for ABTS transformation products. ND, not determined. Blanks, no corresponding values available.
